# A probabilistic classifier for olfactory receptor pseudogenes

**DOI:** 10.1186/1471-2105-7-393

**Published:** 2006-08-29

**Authors:** Idan Menashe, Ronny Aloni, Doron Lancet

**Affiliations:** 1 Department of Molecular Genetics and the Crown Human Genome Center, The Weizmann Institute of Science, Rehovot 76100, Israel

## Abstract

**Background:**

Olfactory receptors (ORs), the largest mammalian gene superfamily (900–1400 genes), has >50% pseudogenes in humans. While most of these inactive genes are identified via coding frame (nonsense) disruptions, seemingly intact genes may also be inactive due to other deleterious (missense) mutations. An ultimate assessment of the actual size of the functional human OR repertoire thus requires an accurate distinction between genes and pseudogenes.

**Results:**

To characterize inactive ORs with intact open reading frame, we have developed a probabilistic Classifier for Olfactory Receptor Pseudogenes (CORP). This algorithm is based on deviations from a functionally crucial consensus, constituting sixty highly conserved positions identified by a comparison of two evolutionarily-constrained OR repertoires (mouse and dog) with a small pseudogene fraction. We used a logistic regression analysis to assign appropriate coefficients to the conserved position and thus achieving maximal separation between active and inactive ORs. Consequently, the algorithms identified only 5% of the mouse functional ORs as pseudogenes, setting an upper limit of 0.05 to the false positive detection. Finally we used this algorithm to classify the 384 purportedly intact human OR genes. Of these, 135 were predicted as likely encoding non-functional proteins, and 38 were segregating between active and inactive forms due to missense polymorphisms.

**Conclusion:**

We demonstrated that the CORP algorithm is capable to distinguish between functional and non-functional OR genes with high precision even when the encoded protein would differ by a single amino acid. Using the CORP algorithm, we predict that ~70% of human OR genes are likely non-functional pseudogenes, a much higher number than hitherto suspected. The method we present may be employed for better annotation of inactive members in other gene families as well.

CORP algorithm is available at:

## Background

Pseudogenes, non-functional gene relics, are highly abundant genome-wide, with an estimated count of at least ~20,000 in the human genome [1,2]. A majority of these (~70%), are processed pseudogenes generated by reverse transcription of mRNAs followed by random genomic integration and thus, resulting in promoter region loss. The remainder non-processed pseudogenes are the result of gene duplication followed by mutational inactivation of one of the redundant copies. Pseudogenes are typically under represented and poorly annotated in the public genome databases. This is because they are considered less interesting, are less easily detected by gene finding programs due to short open reading frames and because some are minimally disrupted and are mistaken for intact genes. Although, pseudogenes do not have evident molecular function, accurate annotation of these interesting genomic loci is valuable for many evolutionary and genetic studies. Consequently new methods are needed for better pseudogenes classification.

Olfaction, the sense of smell, is a versatile and sensitive mechanism for detecting volatile odorous molecules (odorants). Many organisms rely on olfactory cues for a wide range of activities such as food acquisition, reproduction, migration and predator alarming. Accordingly, the olfactory system is capable of detecting and discriminating thousands of low molecular mass compounds. The remarkable sensitivity and specificity of the olfactory system is mediated by olfactory receptors (ORs) [3-5] that are expresses in the olfactory epithelium. OR genes constitute the largest gene superfamily in the mammalian genome, comprising 900–1400 genes [6-10]. This remarkable repertoire, which is essential for chemosensory acuity has evolved through genomic duplications followed by sequence diversification. In the course of recent primate evolution (~10–20 million years) considerable loss of OR genes has taken place, probably as a result of relaxed selective pressure, as species became less dependent on olfactory cues [11,12]. As a result, more than a half of the human ORs are currently annotated as non-processed pseudogenes, containing 1–20 frame-disrupting mutations [7,9]. It is however likely that some seemingly intact genes are in fact non-functional due to missense deleterious mutations emerging due to the same evolutionary process. In this article OR pseudogenes denote such cases that disallow the production of a functional protein, irrespective of transcription status. Some OR pseudogenes may have a stable transcript [13]but in other cases their mRNA may undergo nonsense-mediated decay [14].

Precise characterization of these inactive OR genes is essential for various genetic and functional studies [15-17]. To this end, we have developed a probabilistic Classifier for Olfactory Receptor Pseudogenes (CORP), which quantitatively assesses the probability of an OR gene to be inactive based on the deviation of its inferred protein sequence from a functionally crucial consensus. CORP demonstrated remarkable discrimination, and predicts that >1/3 of the human ORs hitherto considered intact are likely non-functional, some of which show intact-pseudogene segregation in the human population.

## Results

CORP is a probabilistic algorithm that distinguishes between functional and non-functional OR genes (Additional file 1). It is based on the notion that functionally crucial residues are subject to strong selective constraints in active genes, and hence are highly conserved evolutionary. Once the constraint is removed, deleterious mutations are accumulated at these critical positions by neutral drift. Thus, the extent of deviation from a consensus sequence might be a good indicator of the functional status of an OR gene. The algorithm encompasses three consecutive modules: (i) construction of a conservation matrix for constrained OR positions in mouse and dog intact OR learning set, based on the SIFT algorithm [18]; (ii) modifying the conservation matrix by ascribing enhanced weights to position that better distinguish human OR pseudogenes from intact ORs in the other mammals; and (iii) in a test set of intact human genes, computing the cumulative deviation from the weighted conservation matrix, thus assessing the probability of these OR genes to be non-functional.

### Identifying putative functionally crucial residues in OR genes

**Figure 1:**
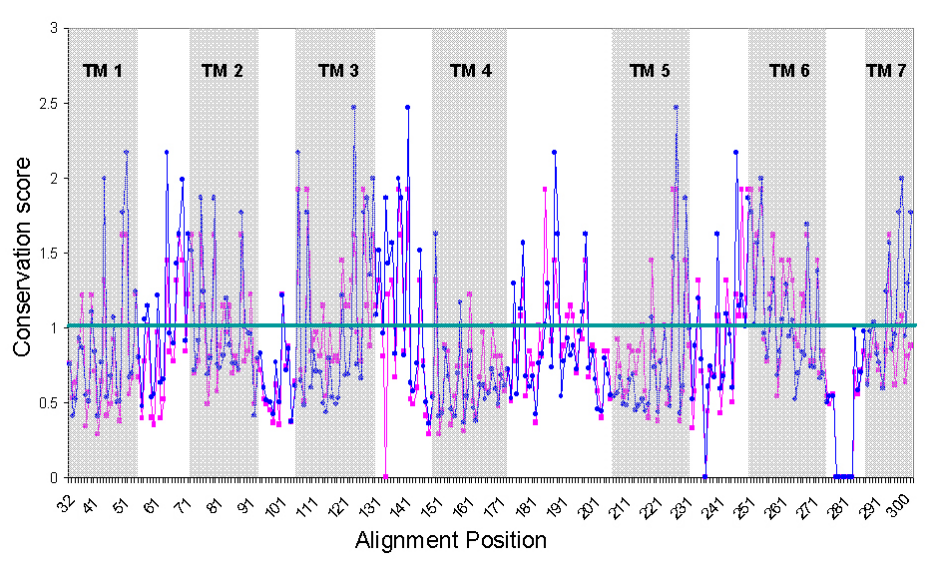
**Positional conservation along OR sequences**. Positions above the horizontal line are such that exceed the conservation score threshold (*P *< 0.05 of chi square and FDR correction) for orthologous pairs (75 positions, Pink) and for paralogous pairs (96 positions, Blue). The overlapping conservation core set includes 65 positions. The conservation scores are normalized per the statistical significance threshold for each of the two sets.

For the characterization of the functionally crucial consensus we assumed: (i) that all ORs have a common ancestral origin [7,10]and hence the most conserved residues in a multiple alignment comparison would be those that are subjected to the strongest functional constraints; (ii) that residue conservation likely represents functional or structural aspects such as signal transduction processes or disulphide bridges shared by all ORs. Accordingly, we analyzed OR genes of dog and mouse, two macrosmatic mammals that still heavily rely on their sense of smell for survival. Intact OR genes in these species are likely to be under strong evolutionary pressure which would tend to eliminate deleterious mutations. We identified residues that were significantly more conserved than expected (*P *< 0.05) both in orthologs and in paralogs (Fig. 1). This was aimed to eliminate positions conserved only in orthologous pairs and not among paralogs, which are candidate contact residues for odorant ligands [19,20]. Also eliminated are residues that are highly conserved in only one of the species (species-specific conservation).

**Figure 2:**
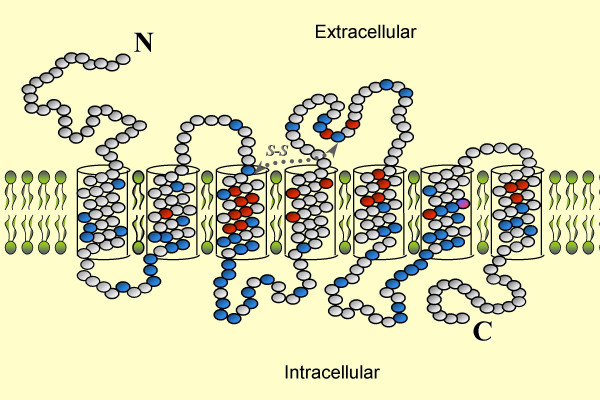
**Localization of conservation core residues in the framework of a 2-dimensional OR diagram**. The inferred locations 65 conservation core residues are shown in blue. For reference the 22 highly variable putative OR complementarity determining residues (CDRs) [19] are also show in red.

Based on this analysis (Fig. 1), 65 positions were found to be highly conserved both among orthologs and paralogs, hence constructing a conservation core which might be related to functionalities shared by all or most ORs. Inspection of the relative position of these conserved residues along the inferred transmembrane helix topology of the OR protein [19] revealed a trend towards an intracellular localization (Fig. 2). This finding supports other studies indicating higher amino acid conservation in the intracellular portion of OR genes [9,21], related to interaction with downstream transduction molecules.

**Figure 3:**
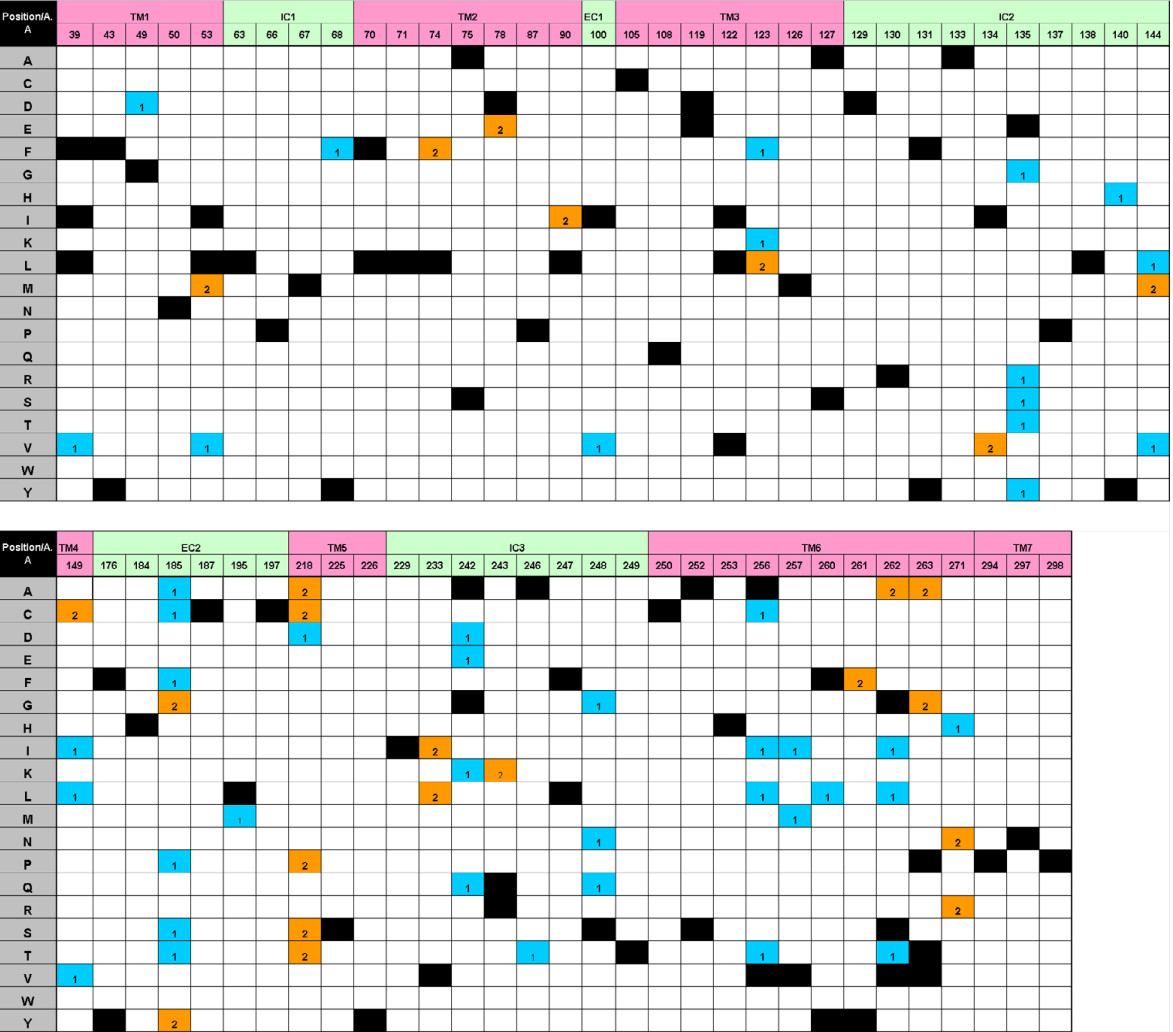
**A SIFT matrix for the OR conservation core residues**. Amino acid indicators (rows) for each of the 65 conservation core positions (columns) are colored if they were characterized by the SIFT algorithm as tolerant for class I ORs (blue), class II ORs (orange) or both classes (black).

To distinguish between tolerant and deleterious amino acids in the conservation core, we employed the SIFT algorithm [18], a sequence homology-based tool that predicts whether an amino acid substitution would have a phenotypic effect. The same dog and mouse intact OR dataset was used, and SIFT was applied separately to class I and class II ORs [6,8] to accommodate class-specific amino acid preferences. Consequently, a class-related SIFT matrix for OR genes was constructed (Fig. 3).

**Figure 4:**
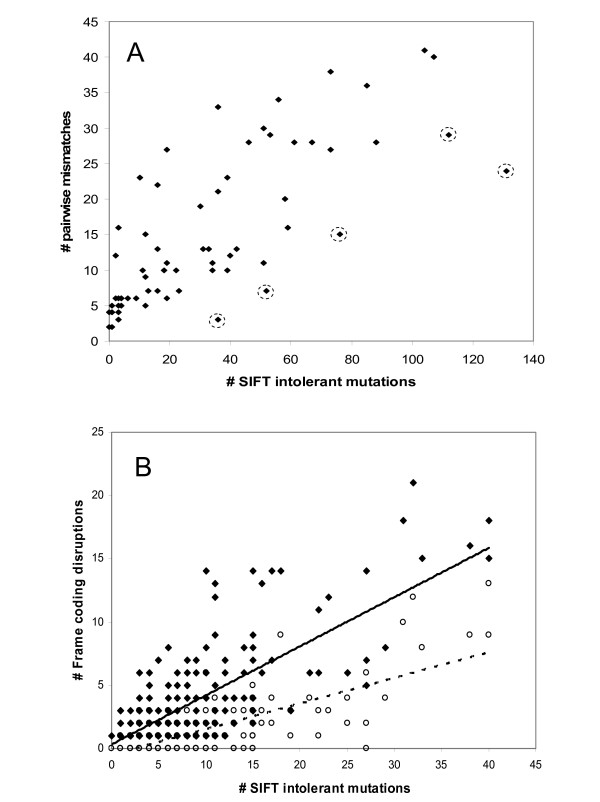
**SIFT matrix validation**. A. The number of SIFT intolerant mutations in each conserved positions (X axis) are plotted vs. their corresponding pair-wise mismatches count of the positional conservation score calculation. Except the large differences in five positions (49, 123, 149, 185 and 262 marked in circles) there is a high agreement between the two methods (r = 0.73 *P *< 10^-5^). (B) The number of SIFT intolerant mutations (X axis) in each of 245 human OR pseudogenes are plotted against the number of coding frame disruptions in their sequences (filled squares) Overall, a high positive correlation is observed even when only frame disruptions between TM1-TM7 are considered (empty circles) (r = 0.75 *P *< 10^-5^, r = 0.73 *P *< 10^-5 ^respectively). Thus, the data in these plots suggests that the SIFT matrix is a good indicator for deviation from selective constraints.

To validate the SIFT matrix results, we examined its agreement with the above pairwise conservation core analysis. For each of the 65 conserved positions, SIFT-intolerant mutations were counted in both mouse and dog intact ORs and the results were compared to their corresponding pairwise conservation scores. Overall, a good agreement was seen (r = 0.73, *P *< 10^-5^) (Fig. 4A). In five positions we observed large differences in the intolerant mutation frequency between the two methods (Fig. 4A) and hence conservatively removed these positions from further analysis.

A key hypothesis of the present study is that a protein's cumulative SIFT intolerance score is a predictor of its pseudogene status. We used the set of 245 non-redundant (< 80% similarity) full-length human OR pseudogenes to obtain support for this notion. For each OR pseudogene the total number of SIFT-intolerant amino acids was compared to the number of frame disruptions (nonsense and in/del mutations). We observed high correlation between these two types of deleterious sequence alterations (r = 0.75, *P *< 10^-5^) (Fig. 4B), suggesting that the cumulative SIFT deviation is an adequate indicator for pseudogenizing sequence disruptions. We also investigated the effect of the position of the coding frame disruption. For this, we reanalyzed the data with only considering frame disruptions between TM1-TM7, as the amino and carboxy termini are the most likely to be dispensable in shortened open reading frame (Fig. 4B). The results show that the observed correlation is not significantly changed.

### Discriminating functional from non-functional ORs

The central goal of this study is to predict whether an OR is functional or not according to its deviation form a protein consensus sequence. For that we used the resulted SIFT matrix to generate a binary vector for each OR gene, indicating a tolerant (0) or intolerant (1) amino acids along the 60 conserved position. To achieve the best separation between presumed active and inactive OR genes, these vectors were subjected to a logistic regression analysis [22], a supervised learning-based classification routine.

The training step of the logistic regression procedure is aimed at computing weighting coefficients for the predictor variables that would subsequently produce likelihood score for belonging to one of the two classes. Here, functional genes were represented by a non-redundant set (< 80% similarity) of 598 mouse intact OR genes, and non-functional genes by 295 human OR pseudogenes. We considered genes with a pseudogene likelihood score *ψ*_*L *_≤ 0.5 as functional and genes with *ψ*_*L *_> 0.5 as pseudogenes. The resulting logistic model correctly characterizing 96.3% of the learning set intact ORs and 69.8% of its human OR pseudogenes (*χ*^2 ^= 541.5, *P *< 10^-8^). We further performed a cross-validation jackknifing analysis, whereby the original training set of 893 genes (both intact and inactive) was divided into 8 equal groups, and learning was performed for different 7/8 subsets, followed by testing on the remained 1/8. Correct identification was obtained for 65% ± 7% of the human pseudogenes and 95% ± 3% of mouse intact genes (P < 10^-8^) (see Additional file 2).

Subsequently, we used the derived logistic model weight parameters to assess the propensity of human OR genes, nominally classified as functional, to encode non-functional proteins. The test set constituted 384 human OR genes with full length open reading frame (> 280 amino acids and including all 7TM regions) labeled as intact in the HORDE database [23]. The procedure resulted in the re-classification of 98 purportedly intact OR genes as non-functional (see Additional file 2). Taking into account the false positive (~5%) and false negative (~35%) values of our logistic model in correctly identifying true pseudogenes (see above), the extrapolated number of intact human ORs that likely encode non-functional proteins is expected to be ~135. This brings the total potential number of human OR pseudogenes to ~70%. Recently, Gilad et al [24] have examined the evolution of OR genes in primates by a genomic comparisons of human to chimpanzee (*Pan troglodytes*). Among other things, they assessed the rate at which neutral gene disruptions accumulate in human OR genes. This led to a subsequent estimation that ~135 human intact OR genes evolve under no selective constraints [24] and Y. Gilad, private communication), which is in agreement with our inference. Thus, both analyses suggest that while a large group of pseudogenes have both missense functional disruptions as well as in-frame stop codons, a significant number of genes (which is in agreement with statistically-based expectations) have only missense-induced loss of function, without coding frame disruptions.

We also utilized CORP to analyze the recently published chimpanzee OR subgenome [24]. Consequently, of 279 chimpanzee full-length intact OR genes, 59 were predicted to be non-functional. The fraction of putatively inactive ORs with intact open reading frame in chimpanzee (0.204) was slightly smaller than observed in human (0.255), as expected according to the similar difference in the frame-disrupted pseudogenes of these species [24]. Such differences suggest that the chimpanzee OR genes evolved under stronger selective constrains than the human OR genes since the speciation of these two higher apes 5–10 million years ago.

### Identifying OR segregating pseudogenes

CORP could also be utilized to predict the functional status of allelic variants in OR genes. In this realm, nonsynonymous SNPs that exchange between tolerant and intolerant amino acids in a highly conserved position may result in segregation between active and inactive alleles. To examine this, we performed a database search for missense SNPs at the 60 highly conserved sequence positions of the OR conservation core of all intact genes. A total of 91 such SNPs were identified in 71 intact OR genes. To assess the potential functional impact of the polymorphisms in these genes, we applied all possible allelic states of these ORs to CORP. Consequently, we found 30 human OR genes segregated between active and inactive states in the human population, arising from mutation-like variations at highly conserved positions. Another 16 ORs that were among the 98 ORs already annotated as non-functional according to other fixed intolerant amino acid substitutions did not change their functional status due to these SNPs. Conversely, 25 ORs were predicted as functional in all their allelic states despite the potentially damaging SNPs in their sequences. Notably, due to the false negative rate of pseudogene identification of our algorithm (~0.3), it is likely that it failed in detecting functional segregation in additional 8 genes. Thereby, it brings the current number of missense segregating OR genes to 38 which more than doubling the known count of such important human genetic variation loci [16].

## Discussion

In this paper we present the CORP algorithm that was designed to tackle an important dilemma of many functional genetic studies: is a gene with an intact ORF necessarily functional? The answer is clearly negative, as mutations in promoters or other regulatory regions as well as changes in crucial protein residues may impair the gene's activity without any obvious sequence disruption. This issue is particularly relevant for human OR genes, a majority of which lost their function in recent human history [11]. Using CORP we evaluated the probability of OR genes to encode an active protein by examining their deviation from an OR functionally-crucial sequence consensus. It is important to note that CORP does not consider the functional consequence of each amino acid substitutions in isolation, but rather the overall number and conservation level of the positions with intolerant mutations. Thus, the resulting pseudogene likelihood score (*ψ*_*L*_) is a reflection of the evolutionary status of the relevant OR gene, a score that is demonstrated here to be a very good predictor of functional status. Since both the conservation core and SIFT matrix were characterized probabilistically, some false positives signals may accrue. Such inaccuracies become less significant through the use of logistic regression analysis that takes into account many other variables. An exception could be an OR that deviates from the conservation core by accumulating so called intolerant mutation to acquire another function not yet identified.

A key parameter in this algorithm is the accurate characterization of potential deleterious amino acid substitutions in highly constrained positions along the OR protein sequence. Conserved sequence motifs of OR genes were previously characterized in various studies [9,21]. However, the delineation of these conservation elements was based on human OR genes, many of which have evolve under minimal selective constraints [12,25]. Therefore, these sequence motifs may not accurately reflect the functionally crucial positions. Another study that used a comparison of two genome assemblies of the mouse to characterize conserved motifs in OR genes [26] is also inadequate for the present purpose, since its invariable motifs are likely masked by species-specific conservation. In contrast, we have constructed an OR gene conservation profile by comparing both OR orthologs and paralogs of mouse and dog. These two species still rely on their sense of smell for survival, augmenting the likelihood of positional conservation. Moreover, these two mammals are sufficiently divergent (~100 Mya) so as to allow better distinction between conserved and variable residues. Therefore it is likely that the resulted conservation core is a good reflection of the functionally crucial mammalian OR positions.

**Figure 5:**
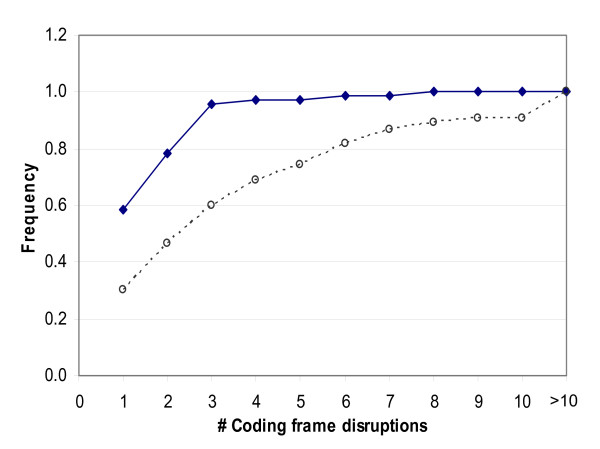
**Frame disruption counts of human OR pseudogenes**. The cumulative frequencies of OR pseudogenes with respect to their number of coding frame disruptions. Continuous line, ORs that are annotated by CORP as 'functional'; Broken line, ORs that are annotated by CORP as 'non-functional'.

The CORP algorithm is better in correctly identifying functional genes (95% success) than in predicting the inactivation of frame-disrupted pseudogenes (65% success). The failure to identify ~1/3 of the pseudogenes as non-functional is rationalized by the observation that the large majority (>95%) of the misclassified pseudogenes had only ≤ 3 frame disruptions in their sequences suggesting that they are recently-formed pseudogenes (Fig. 5). Such recently-formed pseudogenes may not have had time to sufficiently deviate from their conservation core.

**Figure 6:**
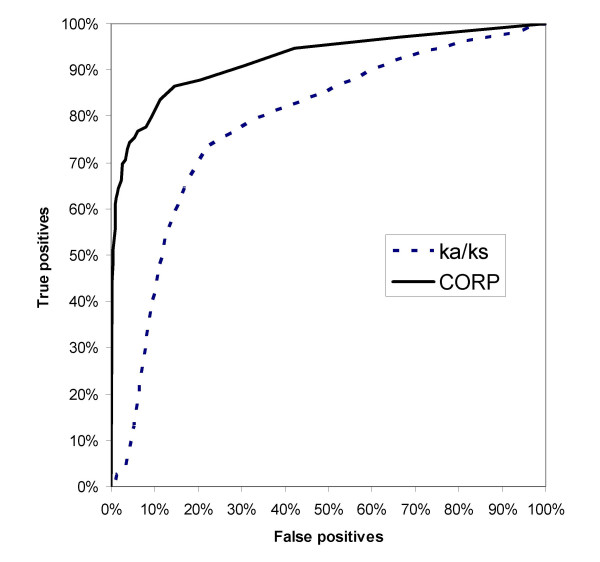
**Receiver operating curve for CORP and Ka/Ks**. The OR pseudogene classification efficiency is indicated by the false positive/true positive ratio. The larger area under the continuous line (93.7% vs. 84.4%) suggests that our method performs better than the Ka/Ks method (solid line) in OR pseudogene classification (A classifier which picks pseudogenes at random would result in a line along the x = y diagonal).

A previous study [2] assessed the conservation level of a gene via the Ka/Ks ratio according to its divergence from its inferred ancestral sequence. The sequence in question is compared to its two closest homologs (one ortholog and one paralog). A low value of Ka/Ks is taken as indicative of Darwinian purifying selection, hence of its functional importance. Applying this method to our training dataset revealed that it correctly identified 77% of the human pseudogenes and 74% of mouse intact genes. While this method performs slightly better in detecting true pseudogenes (67% in our method), it was significantly worse in identifying intact genes (95% in our method). Furthermore, the receiver operating characteristic (ROC) curves were compared for both methods (Fig. 6), indicating a significant advantage of our method.

Another method [27] compares the query sequence to a consensus motif from the Pfam database [28]and calculates whether the deviations from the motif are consistent with a neutral drift model. This algorithm (PSILC), similarly to ours, is based on sequence conservation signals. However, because it utilizes a specific Pfam domain (7TM1) from which ORs deviate considerably it classifies a large majority of intact ORs as pseudogenes. This situation could potentially be improved by a future definition of an OR-specific 7TM Pfam domain. Another potential problem with the application of PSILC to OR sequences is that OR genes are subjected to positive selection [25,29], which may lead to the misclassification of functional genes as pseudogenes [27]. The new version of PSILC which addresses this issue (R. Durbin, private communication) could alleviate this problem. In summary, we have demonstrated that the CORP algorithm is an effective means for *in silico *OR pseudogene identification. It is likely that the same procedure will be applicable to other gene families with similar evolutionary features (e.g. taste or vomeronasal receptor genes). In contrast, in cases of small gene families or single genes it might be preferable to use one of the other existing pseudogene annotation methods.

The ultimate validation for CORP would be experimental examination of the activity of putatively active and inactive OR genes by expression methodologies. Recently, Gaillard et. al [30] demonstrated, that individual amino acid substitutions can abolish the function of particular OR gene. In this experiment they examined the activity of OR 912–93 of several species (OR5G1P in human) and found that it is inactive in orangutan and human despite their intact open reading frame (in human they corrected the existing single in-frame stop codon). Applying the OR sequence of these two species to CORP revealed that both of them were predicted to be non-functional with *ψ*_*L *_= 0.76 and *ψ*_*L *_= 0.67 for human and orangutan respectively. In contrast, the sequences of the active ORs in the other 6 species from this study received very low pseudogene likelihoods scores by our method (an average of *ψ*_*L *_= 0.06) suggesting that they are functional. Interestingly, the function of the two inactive receptors was restored by restoration of the highly conserved Arginine of the DRY motif (located in the interface of TM3 and the 2^nd ^intracellular loop) which is common to many GPCRs and is one of the 60 conserved residues in our conservation matrix. When we introduced the same His-> Arg (orangutan) and Cys->Arg (human) correction to the OR sequences of these species, they were predicted as functional by our algorithm, with pseudogene likelihoods scores of *ψ*_*L *_= 0.15 and *ψ*_*L *_= 0.10 for human and orangutan respectively. This demonstrates the ability of CORP to distinguish between functional and non-functional ORs even if they differ by only one amino acid residue, and provides a limited experimental validation. Despite this supporting evidence for the validity of our algorithm, further studies would help to assess and improve the prediction efficacy of this algorithm.

The validation of functional activity could be based on a number of roles ascribed to OR proteins. The most widely used of these assays is odorant responses [15,17], but other functional roles include plasma membrane targeting [31], protein-mediate negative feedback mechanism that underlies clonal exclusion of OR expression [32,33] as well as axonal guidance in olfactory bulb glomerular targeting [34]. Obviously, an OR may become inactivated by mutations at sites related to one or more of the above functions, in other words inactive ORs may still show undisturbed odorant binding. An advantage of the presently proposed sequence-based functional classification is that it is global, namely will show a high value of *Ψ*_*L *_irrespective of the site or mode of inactivation.

Another major benefit of CORP is its ability to differentiate between functional and non-functional alleles. Here we used this capacity to predict the potential dichotomous functional status of 30 OR allele pairs in the human population. These more than double the known count of OR segregating pseudogenes (SPGs) in the human genome [16], providing additional ground for future genetic studies [35]. Interestingly, 15 of these segregating OR loci included a polymorphism in the conserved Arg_130_. This residue is part of the highly conserved MAYDRY motif [36]. The relatively high number of polymorphisms in Arg_130 _has been previously attributed to the suggestion that it is less functionally important than its neighboring conserved residues (e.g. A_129 _and M_126_) and hence is less constrained by evolutionary selection [26]. However, in our logistic regression analysis this residue received the highest coefficient weight in the comparison of functional and non-functional OR genes, thus suggesting that other biological mechanisms are responsible to the highly polymorphic nature of this residue.

## Conclusion

In this paper we present CORP, a probabilistic algorithm which distinguishes between functional and non-functional OR genes with high precision (Additional file 1). This novel method suggests that the degree of human OR repertoire diminution is considerably higher than thus far been suspected. We demonstrate that CORP can predict, in some cases, the functional consequences of single amino acid substitutions, crucial information for genetic and functional studies. The method and data presented contribute to the improved annotation of pseudogenes, thus helping to further understand these important genomic relics.

## Methods

### OR gene dataset construction

The initial data set comprised 1913 full-length (>280 amino acids) OR protein sequences. These included 753 human genes from build 40 of the HORDE database [23], 1039 mouse [19] and 121 dog [6] OR genes with full-length open reading frame with up to two frame disruptions. Each of these sequences was aligned to a well-curated multiple alignment of mouse and human ORs [19], and the amino acid positions enumerated from 1 to 301 as per the original multiple alignment. Pseudogenes were regarded as genes with at least one frame disrupting mutation between the initiating Methionine and amino acid number 280. The alignments were constructed using Clustal X [37] in a profile alignment mode, with default parameters. Sequences too disrupted to be aligned were discarded. The final data set contained an alignment of 1039, 753 and 83, mouse, human and dog sequences respectively.

We used a previous definition based on mutual best hit [6] for identifying 83 pairs of mouse-dog full-length orthologous OR sequences. The 433 mouse OR paralog pairs were retrieved from [19].

### Calculation of positional conservation scores

The positional conservation scores were calculated separately for pairwise alignments of 83 mouse-dog OR orthologs, and for a similar alignments of 433 mouse OR paralogous pairs. Only residues between TM1 and TM7 (positions 32–301) of the OR sequences were considered, due to poor alignment in the N-terminal and C-terminal ends of the protein. The conservation scores at each residue position *i *was calculated as:

C(i)=−log⁡m(i)n(i)     (1)
 MathType@MTEF@5@5@+=feaafiart1ev1aaatCvAUfKttLearuWrP9MDH5MBPbIqV92AaeXatLxBI9gBaebbnrfifHhDYfgasaacH8akY=wiFfYdH8Gipec8Eeeu0xXdbba9frFj0=OqFfea0dXdd9vqai=hGuQ8kuc9pgc9s8qqaq=dirpe0xb9q8qiLsFr0=vr0=vr0dc8meaabaqaciaacaGaaeqabaqabeGadaaakeaacqWGdbWqcqGGOaakcqWGPbqAcqGGPaqkcqGH9aqpcqGHsislcyGGSbaBcqGGVbWBcqGGNbWzdaWcaaqaaiabd2gaTjabcIcaOiabdMgaPjabcMcaPaqaaiabd6gaUjabcIcaOiabdMgaPjabcMcaPaaacaWLjaGaaCzcamaabmGabaGaeGymaedacaGLOaGaayzkaaaaaa@438A@

where *m(i) *is the number of mismatches between pairs at position *i *and *n(i) *is the total number of comparisons at the same positions. The statistical significance of the positional conservation score (*C(i)*) was assessed by a one-sided chi-square test with one degree of freedom, using the dataset average conservation score as a reference. Finally, we applied a false detection rate (FDR) analysis [38] to eliminate possible false positives due to multiple tests.

To distinguish between tolerant and deleterious amino acids along the OR protein sequence we applied the SIFT algorithm [18] to the dog and mouse OR datasets. Class I and class II ORs were subjected to SIFT separately to eliminate spurious classifications of amino acid substitutions due to the relatively high divergence of these OR classes [7,10].

### Logistic regression analysis

Logistic regression was utilized to distinguish between functional and non-functional OR genes according to their deviations from a functionally crucial consensus. This statistical approach, that attempts to distinguish between classes using the most parsimonious model, employs a set of variables with a known class-identifier to create a model that includes the weighted predictor variables, so as to provide the best separation between classes. Logistic regression was preferred to other statistical methods (e.g. linear discrimination analysis) because it makes no assumptions about the distribution of the independent variables. All the logistic regression analyses in this research were processed by the JMP logistic regression package [39] with its default parameters.

To build the logistic model i.e. to assign the appropriate weight to each conserved position, we used a non-redundant training set (< 80% similarity) of 598 mouse intact OR genes and 295 human OR pseudogenes representing active and inactive OR proteins respectively. According to the resulting model, the probability of belonging to one of the two classes was indicated by a pseudogene likelihood score (*ψ*_*L*_) ranging between 0 (active) and 1 (inactive). Consequently, we considered genes with *ψ*_*L *_≤ 0.5 as functional and genes with *ψ*_*L *_>0.5 as non-functional.

### Databases acquisition of OR SNPs

Missense SNPs in the 60 highly conserved positions were taken from the HORDE databases [23] for all human intact OR genes. SNP information in HORDE is extracted from the NCBI dbSNP database [40] in reference to the OR genomic location. Currently HORDE contains 3395 SNPs of which, 1089 are missense SNPs in intact ORs and hence have a potential functional consequence.

### Ka/Ks calculation

For each of the human and mouse ORs we matched the closest ortholog and paralog according to sequence identity. Then we inferred the ancestral sequence of the gene according to the sequence consensus of this triplet. Subsequently, a value of Ka/Ks was calculated for each gene based on the sequence divergence between the gene and its inferred ancestral sequence. This procedure was performed using GCG package [41].

## Authors' contributions

IM – Conceived and designed the study, carried out the statistical analyses and drafted the ms

RA – Carried out the bioinformatics analyses and helped to draft the ms

DL – Is the PI of the study and prepared the ms.

All authors read and approved the final manuscript.

## Supplementary Material

Additional file 1 The CORP algorithm webpage. Most recent version avaliable at Click here for file

Additional file 2 An excel table with the logistic regression analyses information for the 598 and 245 non-redundant set of mouse intact and human pseudogene ORs respectively as well as for the 384 human and 279 chimpanzee ORs with intact ORF. The information for the various species is given in different worksheets. Human intact gene with missense SNPs in their highly conserved positions are highlighted in orange color and the results of their inferred alleles (marked with a or b at the end of the gene ID) are also indicated. The first column gives the gene ID as appears in the HORDE database [23]. The next 60 columns indicate the tolerant (0) and intolerant (1) residues of each gene according to the SIFT matrix (Fig. 3). The three last columns give the results of the logistic regression analysis. Click here for file
