# Supplementary material for: A probabilistic classifier for olfactory receptor pseudogenes
Source: BMC Bioinformatics. 2006 Aug 29;7:393. doi: 10.1186/1471-2105-7-393 (PMC1599758; doi:10.1186/1471-2105-7-393)
Supplement: Additional file 1 — The CORP algorithm webpage. Most recent version avaliable at [file 1471-2105-7-393-S1.htm]

CORP

**C** lassifier   for   **O** lfactory   **R** eceptor   **P** seudogenes


---

CORP is a probabilistic method for annotation
of olfactory receptor (OR) pseudogenes. The algorithm assesses the probability
of an OR gene with intact open reading frame to encode a non-functional protein
(i.e pseudogene) by examining the deviation of its protein sequences from the OR
functionally crucial consensus. The CORP algorithm was tested on a large dataset
of OR genes and demonstrated excellent distinction between functional and
non-functional ORs.

Link to the article: BMC Bioinformatics -
CORP   
  
  

Paste your OR protein sequence
here:  
   
Select your OR
gene class: **Class I**    
**Class II**  
  
(If you are
not sure what is the Class of your OR sequence, please use "Class II" as a
default).


---

|  |  |  |
| --- | --- | --- |
| Idan Menashe, Ronny Aloni and Doron Lancet Department of Molecular Genetics Weizmann Institute of Science Rehovot, Israel Phone: 972-8-934-2455 Fax: 972-8-934-4487 This file was last modified on | ```  ``` |  |
